# Unraveling the potential carcinogenic risk of bisphenols: a comprehensive network analysis and computational toxicology insights

**DOI:** 10.1186/s40246-026-00948-5

**Published:** 2026-03-14

**Authors:** Tiegang Li, Zheng Yan, Wenyi Zhao, Yufang Hou, Silin Lv, Zifan Zeng, Liu Yang, Mingxuan Zhou, Fang Zhang, Xinyi Ren, Yixin Zhou, Zengni Zhu, Siying Huang, Min Yang

**Affiliations:** 1 State Key Laboratory of Digestive Health, Institute of Materia Medica, Chinese Academy of Medical Sciences and Peking Union Medical College, No. 2 Nanwei Road, 100050 Beijing, China; 2 Beijing Key Laboratory of Key Technologies for Natural Drug Delivery and Novel Formulations, Institute of Materia Medica, Chinese Academy of Medical Sciences and Peking Union Medical College, Beijing, 100050 China; 3 State Key Laboratory of Bioactive Substance and Function of Natural Medicines, Institute of Materia Medica, Chinese Academy of Medical Sciences and Peking Union Medical College, Beijing, 100050 China; 4 MINZU University of China, Beijing, 100081 China

**Keywords:** Bisphenols (BPs), Cancer, Network toxicology, Machine learning, Core targets, Molecular docking, Molecular dynamics simulation

## Abstract

**Purpose:**

Bisphenols (BPs), including bisphenol A (BPA) and its analogs BPB, BPF, BPS, and BPAF, are essential industrial raw materials used in the production of consumer goods but pose significant public health risks. Bisphenols contribute to carcinogenesis due to their endocrine-disrupting properties, particularly in breast cancer. However, the relationship between BPs exposure and putative cancer risk, as well as the underlying molecular mechanisms, remains poorly understood.

**Methods:**

This study employed network toxicology, molecular docking, molecular dynamics simulation, machine learning, and bioinformatics to systematically investigate the molecular mechanisms and potential targets associated with carcinogenic risks of five BPs.

**Results:**

The findings revealed that BPs exposure increases cancer risk by targeting 26 core proteins, including RXRA, AKT2, and CYCS, leading to oxidative stress and modulation of cancer-related signaling pathways such as MAPK, PI3K/AKT, Ras, and VEGF. Molecular docking and dynamics simulations demonstrated stable binding interactions between RXRA and all five BPs. Analysis of the TCM database indicated that *Ginseng*, *Turmeric*, and *Salvia miltiorrhiza* can mitigate BPs-induced cancer risk by targeting these core proteins. Pan-cancer analysis showed that kidney renal clear cell carcinoma (KIRC) and low-grade glioma (LGG) are most strongly associated with BPs exposure. Diagnostic and prognostic models based on core targets exhibited high predictive accuracy, offering valuable clinical decision support. Single-cell sequencing revealed that core targets are primarily localized in immune cells in KIRC and glioma cells in LGG.

**Conclusion:**

This study provides a theoretical foundation for evaluating cancer risk associated with BPs exposure and establishes a novel framework for understanding the pathogenesis and potential therapeutic strategies for environmental pollutants.

**Supplementary Information:**

The online version contains supplementary material available at 10.1186/s40246-026-00948-5.

## Introduction

From ubiquitous plastic containers to thermal receipts, modern life is deeply entwined with polymer-based products. However, this convenience conceals a latent health threat posed by Bisphenol compounds (BPs). These synthetic chemicals, characterized by two phenolic rings connected via carbon or sulfur bridges, form the chemical foundation of high-performance materials such as polycarbonate (PC), Polysulfone (PSU), and polyphenylene oxide (PPO). While essential in the production of durable industrial components and everyday consumer goods, specific BPs—particularly Bisphenol A (BPA) and its analogs (BPB, BPF, BPS, BPAF)—have emerged as potent endocrine-disrupting chemicals (EDCs) [1]. Through dietary ingestion, inhalation of microplastics, and dermal absorption from receipts or packaging, these xenoestrogens infiltrate human systems, acting as silent ‘health assassins’.

Bisphenol A, the first industrially produced and most widely used BPs, has substantial evidence linking its exposure to an increased susceptibility to tumorigenesis [2, 3]. Analysis of the National Health and Nutrition Examination Survey (NHANES) dataset revealed a U-shaped association between BPA exposure and cancer mortality risk, with BPA levels below 1.99 ng/mL correlating with heightened cancer mortality risk [4]. Beyond tumors, studies have also established links between BPA exposure and numerous chronic health conditions, including obesity, diabetes, cardiovascular diseases, chronic respiratory disorders, behavioral issues, and reproductive dysfunctions [5–8]. In response to these health risks, the Chinese government enacted a nationwide ban on the import and sale of BPA-containing baby bottles in 2011. Similarly, the French Law No. 2012-1442, effective January 1, 2015, prohibits the use of BPA in all food-contact packaging materials.

In light of growing public concern over BPA and stricter government regulations, numerous alternative compounds—such as BPB, BPF, BPS, and BPAF—have been introduced to the market. The increasing use of these substitutes in BPA-free products has turned them into emerging environmental contaminants, with detectable concentrations in surface waters ranging from single to hundreds of nanograms per liter [9]. Given their structural and functional similarities to BPA, these substitutes may also pose potential carcinogenic risks. However, epidemiological data and preclinical studies on the human health impacts of these substitutes remain limited. Some studies have suggested that BPs exposure can alter DNA methylation in transposons and breast cancer-related gene promoters CDH1(Cadherin-1), SFN(14-3-3 protein sigma), TNFRSF10C(Tumor necrosis factor receptor superfamily member 10 C), indicating a potential epigenetic role in breast cancer development [10]. Bisphenol B and BPAF exposure has been linked to ovarian morphological disruptions via multifactorial pathological mechanisms, contributing to ovarian lesions through dysregulated mRNA expression profiles [11]. In the hepatocellular carcinoma (HCC) patients, BPs concentrations were ranked in serum > whole blood > urine, showing significant positive correlations with liver dysfunction markers [12]. Nonetheless, the carcinogenicity of these bisphenols and their underlying toxicological mechanisms remain to be fully elucidated.

Traditional toxicological methods are often limited to single-target analyses and isolated experimental models [13, 14]. While this approach can uncover direct interactions between toxicant exposure and biomolecules under specific conditions, it falls short in addressing multi-target synergistic toxicity and cross-organ toxicity networks. In contrast, human disease pathogenesis is a complex process influenced by genetic predispositions, environmental exposures, and other factors, involving dynamic interactions across genes, proteins, and metabolites. Network toxicology, grounded in systems biology, integrates multi-omics data to construct “toxicant-target-disease” multidimensional networks, systematically unraveling the perturbative effects of chemical toxicants on biological systems [15, 16]. This approach not only identifies key molecular targets and pathways but also uncovers interconnections between diverse biological processes. As such, network toxicology provides a research framework that better aligns with the complex pathological reality of environmental toxicant-induced diseases.

This study comprehensively investigated the potential carcinogenic risks and underlying molecular mechanisms of long-term, low-dose exposure to BPs, including BPA, BPB, BPF, BPS, and BPAF. Initially, network toxicology methods were employed to identify the molecular mechanisms and hub target proteins involved in BPs-induced carcinogenesis. Molecular docking and molecular dynamics (MD) simulations were then used to validate the binding potential between BPs and hub target proteins. Pan-cancer survival analysis was conducted to identify the tumor types most significantly affected by BPs exposure. Diagnostic and prognostic models for tumors were developed using machine learning techniques to clarify the roles of hub targets in tumorigenesis and progression. Finally, the expression patterns of hub targets were validated through single-cell RNA sequencing (scRNA-seq) and immunohistochemistry (IHC). These findings offer a comprehensive pan-cancer perspective on BPs-induced carcinogenesis, providing a scientific basis for the pathogenesis and prevention of environmental pollutants.

## Methods

### Chemical structure and toxicity prediction of BPs

The chemical structure information for BPA and its analogs—Bisphenol B (BPB), Bisphenol F (BPF), Bisphenol S (BPS), and Bisphenol AF (BPAF)—was obtained from the PubChem database (https://pubchem.ncbi.nlm.nih.gov/). To evaluate the toxicity of these compounds, the ADMETlab 3.0 platform (https://admetlab3.scbdd.com/), a comprehensive tool for predicting absorption, distribution, metabolism, excretion, and toxicity (ADMET) profiles of chemicals, was used.

### Detection and analysis of BPs in urine

Urinary levels of BPs, including BPA, BPS, and BPF, were retrieved from the NHANES database (https://wwwn.cdc.gov/nchs/nhanes/Default.aspx). Data for BPA spans seven survey cycles from 2003 to 2016, while detection data for BPS and BPF are available for the cycles from 2013 to 2014 and 2015–2016. Urinary concentrations of these BPs were measured using high-performance liquid chromatography coupled with mass spectrometry (HPLC-MS/MS). Detailed urine sample pretreatment procedures, chromatography-mass spectrometry detection parameters, and data analysis methodologies can be accessed on the NHANES official website under the “Laboratory Method Files”. Furthermore, information regarding whether participants have cancer, as well as the type of cancer, was derived from the NHANES Questionnaire Data. Based on the interview data, cancer diagnoses for 28 different types of cancers were obtained. Survival status data for adult NHANES participants were sourced from the National Death Index (NDI) Mortality Data (https://www.cdc.gov/nchs/data-linkage/mortality.htm), with cancer-specific mortality data referring to cases where the Underlying Cause of Death Leading (UCOD_LEADING) was assigned code 2. Kaplan–Meier (KM) survival analysis, using the ‘survival’ package in R, was employed to investigate the correlation between urinary bisphenol concentrations and survival outcomes in cancer patients.

### Collection of potential targets of BPs

To predict the potential molecular targets of BPs, chemical names or SMILES (Simplified Molecular Input Line Entry System) representations were queried in nine online databases: ChEMBL (https://www.ebi.ac.uk/chembl/), CTD (https://ctdbase.org/), GalaxySagittarius-AF (https://galaxy.seoklab.org/), PharmMapper (https://www.lilab-ecust.cn/pharmmapper/index.html), SEA (https://sea.bkslab.org/), STITCH (http://stitch.embl.de/), Super-PRED (https://prediction.charite.de/), SwissTargetPrediction (http://swisstargetprediction.ch/), and TargetNet (http://targetnet.scbdd.com/). The potential targets identified from these databases were aggregated and deduplicated to generate a final target gene set for the BPs.

### Selection of tumor-related molecules

To systematically identify molecular targets associated with putative tumorigenesis, keywords such as “Tumor,” “Cancer,” “Malignancy,” and “Carcinoma” were queried in OMIM (https://www.omim.org/), GeneCards (https://www.genecards.org/), and CTD (https://ctdbase.org/). Following the integration of gene lists from these sources and subsequent deduplication, a curated set of tumor-associated molecular entities was compiled.

### Functional and pathway enrichment analyses

To investigate the potential carcinogenic mechanisms of BPs exposure, Gene Ontology (GO) functional enrichment and Kyoto Encyclopedia of Genes and Genomes (KEGG) pathway enrichment analyses were performed on the 1,112 intersecting genes derived from the shared regulatory targets of BPs and tumor-associated molecules using the ‘clusterProfiler’ package in R. The GO enrichment analysis focused primarily on molecular functions (MF) and biological processes (BP), with terms exhibiting a p.adjust value below 0.05 considered statistically significant.

### Construction of the protein–protein interaction (PPI) network

The 1,112 overlapping genes were uploaded to the STRING database for PPI analysis, with interaction confidence scores set to > 0.9. Disconnected nodes were excluded from the network. The network data in TSV format were exported from STRING and visualized using Cytoscape 3.10.1 software to analyze topological parameters [17]. Topological properties of nodes in the PPI network, including closeness, degree, and betweenness, were calculated using the CentiScaPe 2.2 plugin. Nodes with values exceeding the mean for these topological parameters were identified as critical nodes.

### Screening of hub targets for the putative tumorigenesis induced by BPs

Gene sets linked to the canonical oncogenic pathways (hsa05200) were obtained from the GSEA database (https://www.gsea-msigdb.org/gsea/index.jsp). Overlapping genes from this pathway and the critical nodes identified in the PPI analysis were designated as key factors in BPs-induced carcinogenesis. Subsequently, genes interacting with all the bisphenol analogs (BPA, BPB, BPF, BPS, BPAF) were defined as hub genes. Concurrently, GO and KEGG functional and pathway enrichment analyses were performed to examine the biological roles of these hub targets.

### Molecular docking

To further assess the binding affinity and interaction patterns between the five BPs and their hub targets, molecular docking was performed using AutoDock *Vina* 1.1.2. The SDF (Structure Data File) files of 2D structural small molecule ligands were sourced from the PubChem database and imported into Avogadro 1.2.0 software for geometry optimization and 3D file generation in PDB format. High-resolution protein crystallographic data for the hub targets were retrieved from the RCSB PDB repository (https://www.rcsb.org/). In the absence of experimental structures, AlphaFold-predicted protein models obtained from the UniProt database (https://www.uniprot.org/) were used as structural alternatives. If the protein crystal structure contained endogenous small molecule ligands, the binding pocket was defined around the ligand’s position; otherwise, the grid box covered the entire protein surface as the potential binding interface for docking simulations. Water molecules and original ligands within protein crystal structures were removed using PyMOL 2.6.0 software. Binding affinities (kcal/mol) were calculated to evaluate the stability of ligand-receptor interactions, with lower values indicating more favorable binding. Finally, the binding patterns and interactions of the protein-ligand complexes were visualized using Discovery Studio 2023.

### Molecular dynamics simulation

Using the free and open-source software GROMACS 2024.4, MD simulations were performed on the ligand-receptor complex with the lowest binding energy identified through molecular docking, further validating the stability of the complex. First, the CHARMM36 force field parameters were assigned to the protein molecule and CGenFF force field parameters to the ligand molecule to generate topology files. A water box was then constructed using the TIP3P water model to simulate physiological conditions, and counterions were added to neutralize the net charge. Initial energy minimization was performed, followed by NVT (constant temperature and volume) and NPT (constant pressure and temperature) equilibration. Finally, an 80 ns MD simulation was carried out at 310 K and 1 bar, with trajectory frames recorded every 2 ps.

To evaluate system stability, parameters including RMSD (Root Mean Square Deviation), RMSF (Root Mean Square Fluctuation), Rg (Radius of Gyration), SASA (Solvent Accessible Surface Area), and H-bonds (hydrogen bonds) were computed from the simulated trajectory files and analyzed visually using the ‘ggplot2’ package. Subsequently, the trajectory files corresponding to the equilibrated state within the 70–80 ns range were extracted to calculate the average binding free energy using the MM/GBSA (Molecular Mechanics/Generalized Born Surface Area) and MM/PBSA (Molecular Mechanics/Poisson-Boltzmann Surface Area) methods [18]. The Gibbs free energy landscape (FEL) was generated using Rg and RMSD values to visualize the energy distribution across different conformational states sampled during the MD simulations.

### Predicting potential therapeutic interventions of Traditional Chinese Medicine (TCM) herbs and diet based on hub targets

Next, the hub targets identified in the previous section were input into the CoreMine Medical database (https://coremine.com/medical/?locale=zh_CN) to search for Chinese herbal medicines and dietary interventions that may mitigate BPs exposure. An interaction network diagram linking the hub targets, TCM herbs, and foods was constructed using Cytoscape software. CoreMine Medical utilizes semantic network technology to dynamically connect biomedical entities—including diseases, genes, pharmaceuticals, TCM herbs, and chemical components—enabling researchers to explore statistically validated associations and interactive networks across diseases, therapeutics, and molecular mechanisms.

### Pan-cancer analysis of the hub targets implicated in BPs exposure

Expression profiles of the hub targets related to BPs exposure across 33 pan-cancer types were retrieved from the GEPIA2 database (http://gepia2.cancer-pku.cn/), followed by visualization of the expression heatmaps using the R package ‘ComplexHeatmap’. Univariate Cox regression analysis was then conducted to investigate the prognostic relevance of the hub targets across these tumor types, with data sourced from the TISIDB database (http://cis.hku.hk/TISIDB/), to identify the tumor types most significantly affected by BPs exposure.

### The Cancer Genome Atlas (TCGA) data collection for LGG and KIRC

To further validate the roles of hub targets associated with bisphenol exposure in low-grade glioma (LGG) and kidney renal clear cell carcinoma (KIRC), RNA sequencing expression data in log2(TPM + 1) format and corresponding clinical information were downloaded from the UCSC Xena database (https://xena.ucsc.edu/). The KIRC cohort consists of 610 sequencing samples, including 72 normal and 538 tumor samples, while the LGG cohort includes 534 tumor samples. Given that the LGG cohort consists solely of tumor specimens, the diagnostic model was constructed using only the KIRC data.

### Construction of a KIRC diagnostic model based on the hub targets associated with BPs exposure by six ensemble machine learning algorithms

The KIRC sequencing data were initially divided into training and validation sets at an 8:2 ratio, with stratified sampling to maintain consistent proportions of normal and tumor samples in both sets. Six ensemble machine learning algorithms—Adaptive Boosting (AdaBoost), Gradient Boosting Machine (GBM), Light Gradient Boosting Machine (LightGBM), Random Forest (RF), Categorical Boosting (CatBoost), and Extreme Gradient Boosting (XGBoost)—were used to calculate SHAP (SHapley Additive exPlanations) values for the hub targets, quantifying their contributions to model predictions. Based on the feature importance rankings derived from SHAP values, a recursive feature elimination approach was applied to iteratively reduce the number of features until a significant decline in model performance, such as AUC (Area under the curve) values [19]. This process identified the optimal model and the most critical features for model construction, followed by an evaluation of their diagnostic performance on the test set. All analyses were conducted using Python version 3.10.9.

### Constructing a risk prognosis model for KIRC and LGG based on the hub targets related to BPs exposure

Univariate Cox regression analysis was initially performed to evaluate the prognostic significance of the hub targets associated with BPs exposure in both LGG and KIRC tumors. Forest plots were generated using the “forestplot” package for visualization. Genes with P-values less than 0.05 in the univariate Cox regression analysis were selected for subsequent construction of the prognostic risk model.

A Lasso-Cox proportional hazards regression model was then developed using the ‘glmnet’ package, incorporating these significant prognostic genes to identify the most robust predictors of survival outcomes. The risk scoring formula was subsequently formulated as:

$$\mathrm{R}\mathrm{i}\mathrm{s}\mathrm{k} \, \mathrm{S}\mathrm{c}\mathrm{o}\mathrm{r}\mathrm{e}=\sum_{\mathrm{i}\mathrm{=1}}^{\mathrm{p}}\mathrm{{\beta}}\mathrm{i}\mathrm{*}\mathrm{Xi}$$

where βi represents the Lasso-Cox proportional hazards regression coefficient for gene Xi, and Xi denotes the expression level of the corresponding gene. The prognostic risk score for each patient was calculated using this algorithm, with cohorts dichotomized into high- and low-risk subgroups based on the cohort-specific median cutoff value.

Kaplan–Meier survival analysis was performed using the ‘survminer’ package to evaluate survival differences between high- and low-risk groups, with statistical significance assessed via the log-rank test (*P* < 0.05). The prognostic signature was visualized through a risk stratification plot, illustrating the distribution of risk scores and categorization of survival outcomes across distinct patient subgroups. Receiver Operating Characteristic (ROC) curve analysis for 1-, 3-, and 5-year survival was conducted using the ‘timeROC’ package to assess the predictive performance of the risk score. To evaluate the independent prognostic value of the risk score, both univariate and multivariate Cox proportional hazards regression analyses were performed, incorporating the risk score and clinically relevant covariates. A nomogram, integrating multivariable Cox-derived prognostic factors, was developed using the ‘rms’ package to estimate personalized survival probabilities. Its predictive accuracy was validated through calibration curves and decision curve analysis (DCA).

### Single-cell RNA sequencing (scRNA-seq) data analysis

To investigate the expression patterns of core modeling genes within the tumor microenvironment, a single-cell data analysis was conducted. Single-cell sequencing data for KIRC and LGG were obtained from the GEO database (https://www.ncbi.nlm.nih.gov/geo/), with the corresponding GEO accession numbers being GSE242299 (for KIRC) and GSE182109 (for LGG). The KIRC cohort includes 8 fresh tumor samples and 9 adjacent healthy tissue samples. Data analysis was carried out using the ‘Scanpy’ toolkit in Python, with specific analytical procedures outlined in the original article [20]. The GSE182109 dataset contains scRNA-seq data from 2 LGG cases, with analysis performed using the ‘Seurat’ package in R, following the methods outlined in the original study [21].

### Protein expression analysis of core modeling genes

Protein expression analysis of the core modeling genes was performed in healthy and cancerous tissues from the HPA dataset (http://www.proteinatlas.org/) through IHC staining.

## Results

### Evaluation of physicochemical characteristics and environmental toxicity profiles of BPs

**Fig. 1:**
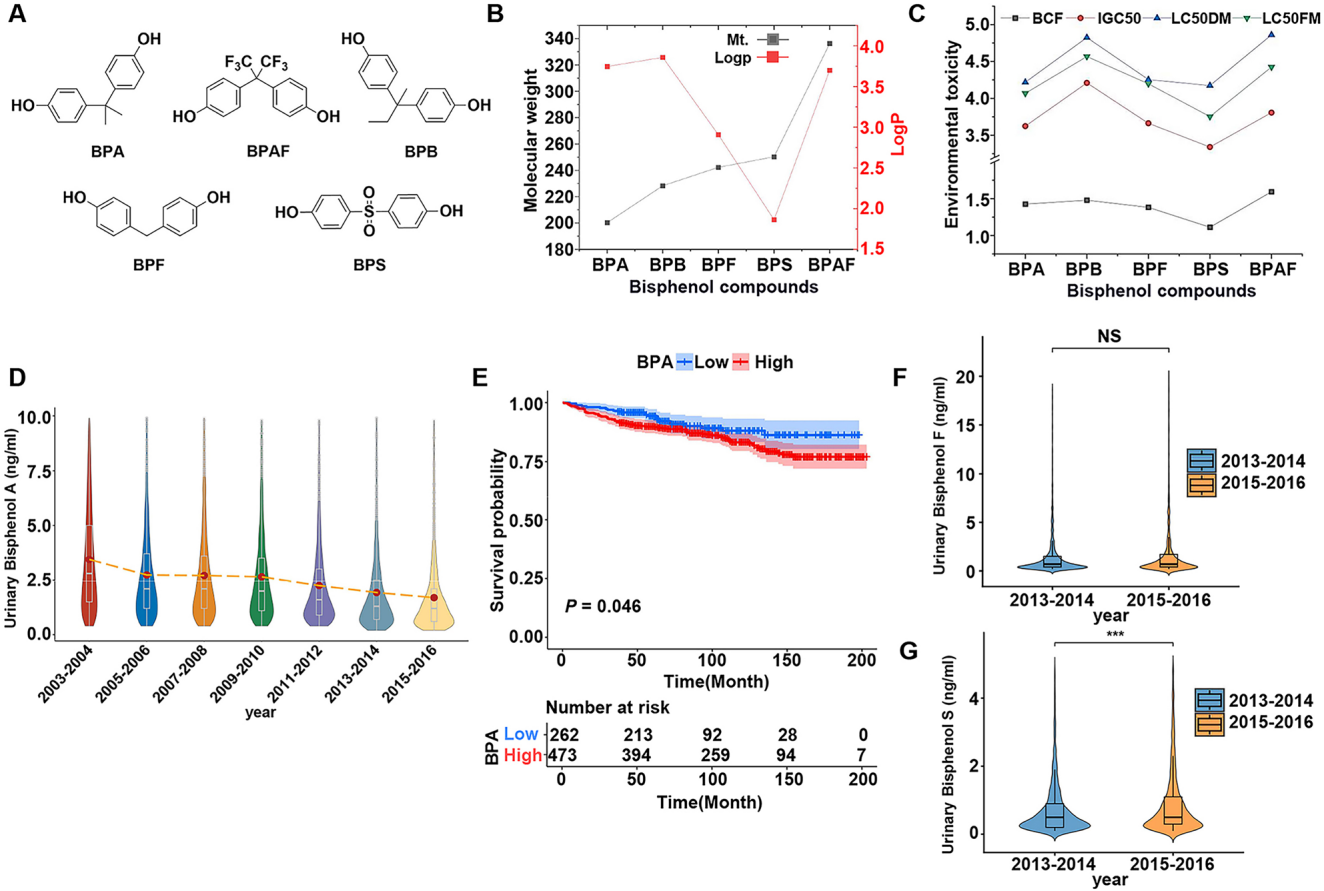
Overview of five types of bisphenol compounds. **A** Chemical structure of bisphenol A and its analogs. **B** Physicochemical properties of BPs: Molecular weight (Mt.) and Log P (Octanol-water partition coefficient). **C** Biotoxicity metrics for 5 BPs: LC50DM: Median lethal concentration in Daphnia magna (48 h); LC50FM: Median lethal concentration in Pimephales promelas (96 h); IGC50: 50% growth inhibition concentration in Tetrahymena pyriformis; BCF: Bioconcentration factor. **D** Levels of Bisphenol A in urine among the U.S. population from the NHANES database, 2003 to 2016. **E** Analysis of the association between urinary Bisphenol A levels and overall survival (OS) using Kaplan–Meier curves in cancer patients from the NHANES database. **F** Levels of Bisphenol F and Bisphenol S in urine among the U.S. population from the NHANES database, 2013 to 2016

The specific overview and chemical structures of BPs are shown in Supplementary Table S1 and Fig. 1A. The physicochemical properties and environmental toxicity of these compounds were systematically predicted using the ADMETlab database. Bisphenol A, BPB, and BPAF exhibit similar lipophilicity-hydrophilicity partition coefficients (*logP*), while BPAF and BPS demonstrate lower *logP* values due to the presence of a strong electron-withdrawing trifluoromethyl group (-CF3) in BPAF and a highly hydrophilic sulfonyl group (-SO2-) in BPS, respectively (Fig. 1B). Similarly, BPS, owing to the sulfonyl group (-SO2-), exhibits the highest environmental toxicity, while the trifluoromethyl group in BPB helps reduce its environmental toxicity to some extent (Fig. 1C).

Although several U.S. states have banned the use of BPA in children’s food containers since 2009, large-scale biomonitoring studies reveal that BPA remains detectable in human urine, suggesting potential bioaccumulation within the human body (Fig. 1D). Moreover, KM survival analyses show a negative correlation between BPA exposure levels and cancer survival outcomes, with higher mortality risks associated with increased urinary BPA concentrations (Fig. 1E). As BPA alternatives such as BPF and BPS become more widely adopted, these bisphenol chemicals have increasingly been detected in human urine (Fig. 1F). Notably, urinary concentrations of BPS show an upward trend with prolonged exposure duration (Fig. 1G).

### Identification of underlying targets of BPs and tumor-associated molecules

**Fig. 2:**
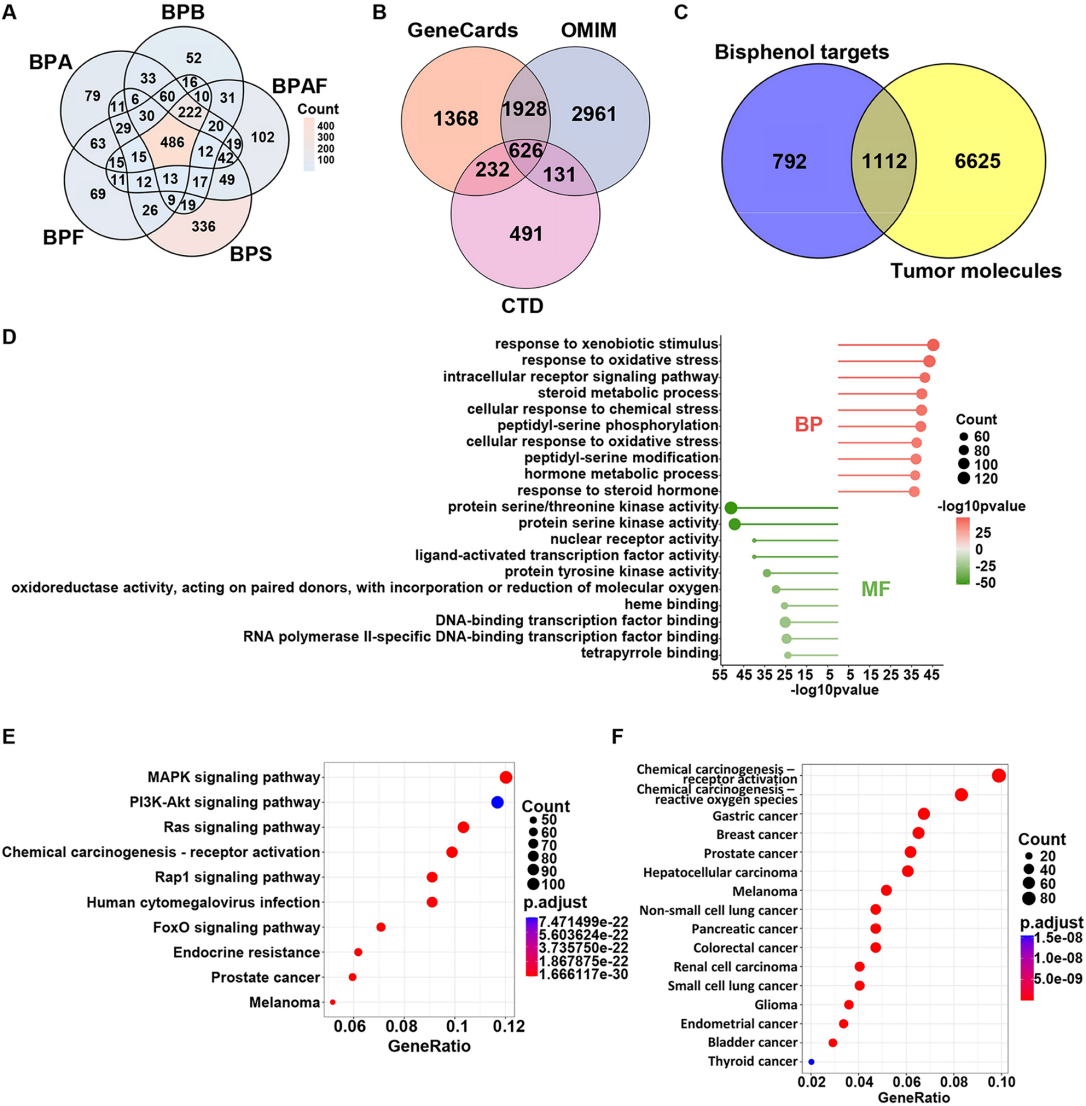
Network toxicology analysis of BPs-induced tumorigenesis risk. **A** Venn diagram of BPs targets. **B** Tumor-associated molecular Venn diagram. **C** Venn diagram of BPs targets and tumor-associated molecules. **D** Bar chart of GO enrichment analysis. **E**, **F** Bubble diagram of KEGG enrichment analysis. *GO* Gene Ontology; *KEGG* Kyoto Encyclopedia of Genes and Genomes; *BP* biological process; *MF* molecular function

Following an analysis of online databases, a total of 1,904 unique candidate target proteins were identified for BPA and its analogs through systematic integration and deduplication of the preliminary results. Specifically, BPA interacted with 1,142 proteins, BPB was associated with 1,026 proteins, BPF mapped to 1,076 proteins, BPS correlated with 1,102 proteins, and BPAF modulated 1,066 proteins. Venn diagram analysis revealed 486 shared target proteins among these compounds (Fig. 2A). Next, 7,737 molecules highly relevant to tumors were identified through database searches. The OMIM database yielded 5,649 molecules, GeneCards compiled 4,154 molecules, and the CTD database reported 1,480 molecules (Fig. 2B). By integrating toxicant-related targets and tumor-associated molecules, 1,112 overlapping targets were identified, potentially implicated in the carcinogenic mechanisms of BPs (Fig. 2C).

To elucidate the biological mechanisms underlying bisphenol exposure in cancer development, GO and KEGG functional and pathway enrichment analyses were conducted on the 1,112 common genes. The top ten BP terms were primarily focused on responses to exogenous stimuli, such as responses to xenobiotic stimulus and oxidative stress, cellular responses to chemical and oxidative stress, and endocrine resistance. Molecular function (MF) was predominantly observed in pathways related to cell proliferation, including protein kinase activities, DNA transcription, and replication (Fig. 2D). Additionally, the top ten most significant pathways in KEGG were concentrated on cell proliferation, chemical carcinogenesis, and tumor-related signaling pathways, including MAPK, PI3K-Akt, Ras, FoxO signaling, chemical carcinogenesis receptor activation, prostate cancer, and melanoma (Fig. 2E). Enrichment analysis based on KEGG also indicated that these genes are significantly associated with 14 types of cancer, suggesting that bisphenol compound exposure may promote tumorigenesis through multiple molecular pathways (Fig. 2F).

### Screening of hub targets for tumorigenesis induced by bisphenol compounds exposure and construction of the PPI network

**Fig. 3:**
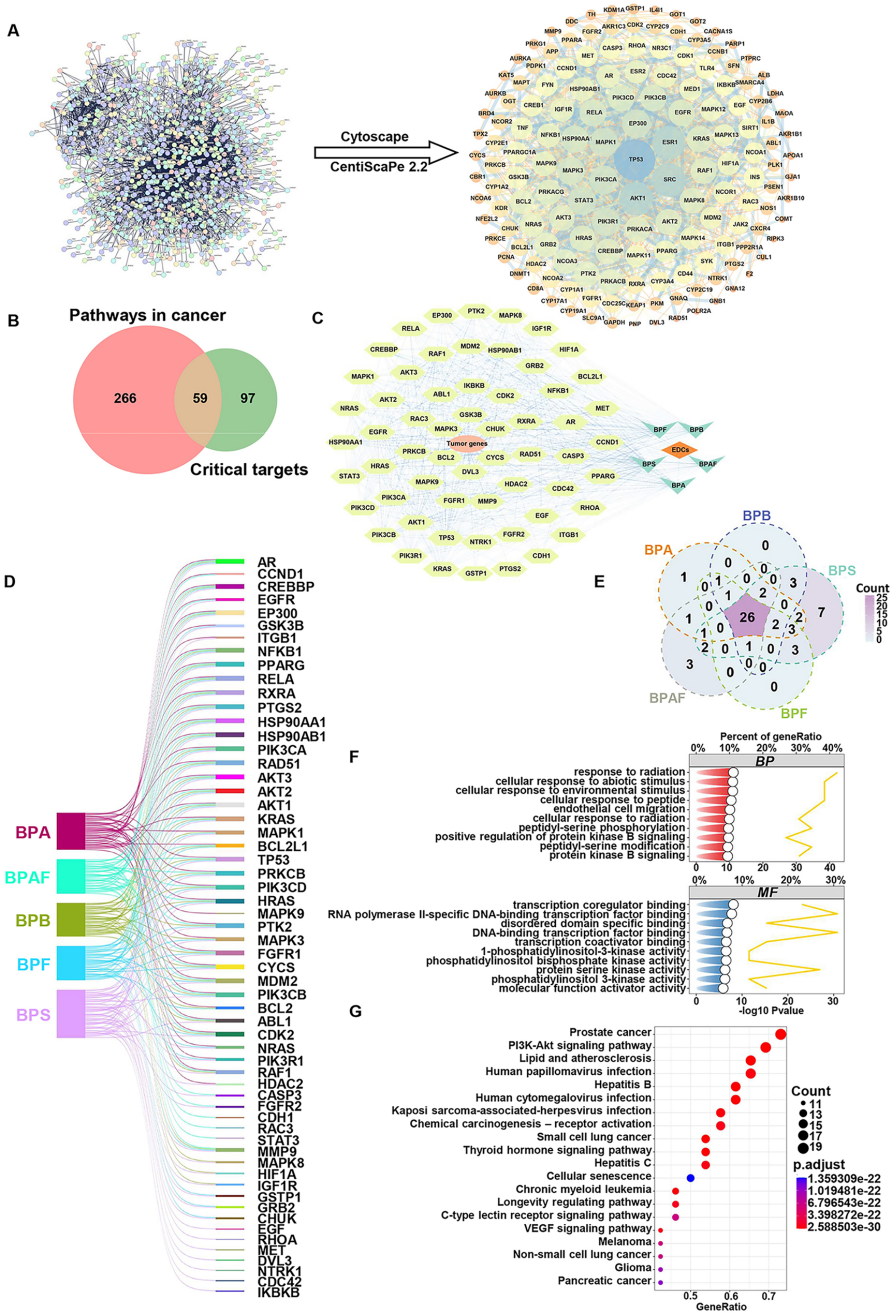
Screening of core targets for BPs exposure-induced tumorigenesis. **A** Protein–protein interaction (PPI) network visualization of the core tumorigenesis-related targets modulated by BPs. **B** Intersection Venn diagram of cancer pathways and critical targets in BPs-induced tumorigenesis. **C** Network of endocrine-disrupting chemicals (EDCs)-BPs-tumor genes-targets, with yellow ellipse nodes indicating targets, orange ellipse nodes for tumor genes, brown diamond nodes for EDCs, and cyan V-shaped nodes for BPs. **D** Sankey diagram of BPs and their potential regulatory targets. **E** Common molecular targets for carcinogenesis induced by five Bisphenols. **F** Comet plot visualization of GO term enrichment. **G** Bubble diagram of KEGG enrichment analysis

The 1,112 potential tumor-inducing targets of BPs were imported into the STRING database. Network parameters were configured to generate an interaction network comprising 894 nodes and 4,180 edges (Fig. 3A). This network was visualized using Cytoscape software, and topological properties of network nodes were calculated using the CentiScaPe 2.2 plugin. Nodes with Closeness ≥ 0.0385, Betweenness ≥ 2,350.0514, and Degree ≥ 9.3512 were identified as critical nodes within the network. After screening the topological parameters, a PPI network consisting of 156 critical nodes and 1,069 edges was obtained (Fig. 3A). The 156 critical targets in the PPI network were then intersected with 325 genes from canonical cancer signaling pathways, resulting in 59 critical genes implicated in BPs-induced putative tumorigenesis (Fig. 3B). Additionally, a network diagram was constructed to illustrate the interactions between endocrine disruptors, bisphenols, tumors, and critical targets (Fig. 3C). Sankey diagram analysis revealed that among these critical targets, 40 interacted with BPA, 36 with BPB, 37 with BPF, 52 with BPS, and 37 with BPAF (Fig. 3D).

Furthermore, Venn diagram analysis identified 26 targets commonly regulated by all five BPs, which are considered hub targets implicated in tumorigenesis induced by bisphenol exposure (Fig. 3E). Enrichment analysis based on GO revealed that these hub genes were significantly enriched in responses to environmental stimuli, endothelial cell migration, protein kinase B signaling, as well as regulation of DNA transcription, translation, and the activity of protein kinases modulating cell proliferation (Fig. 3F). The top 20 KEGG pathways were primarily involved in cancer-related pathways, including prostate cancer, small cell lung cancer, melanoma, non-small cell lung cancer, glioma, pancreatic cancer, PI3K-Akt signaling, VEGF signaling, and chemical carcinogenesis (Fig. 3F).

### Elucidating binding efficacy of Bisphenol A analogs to the hub targets through molecular docking

**Fig. 4:**
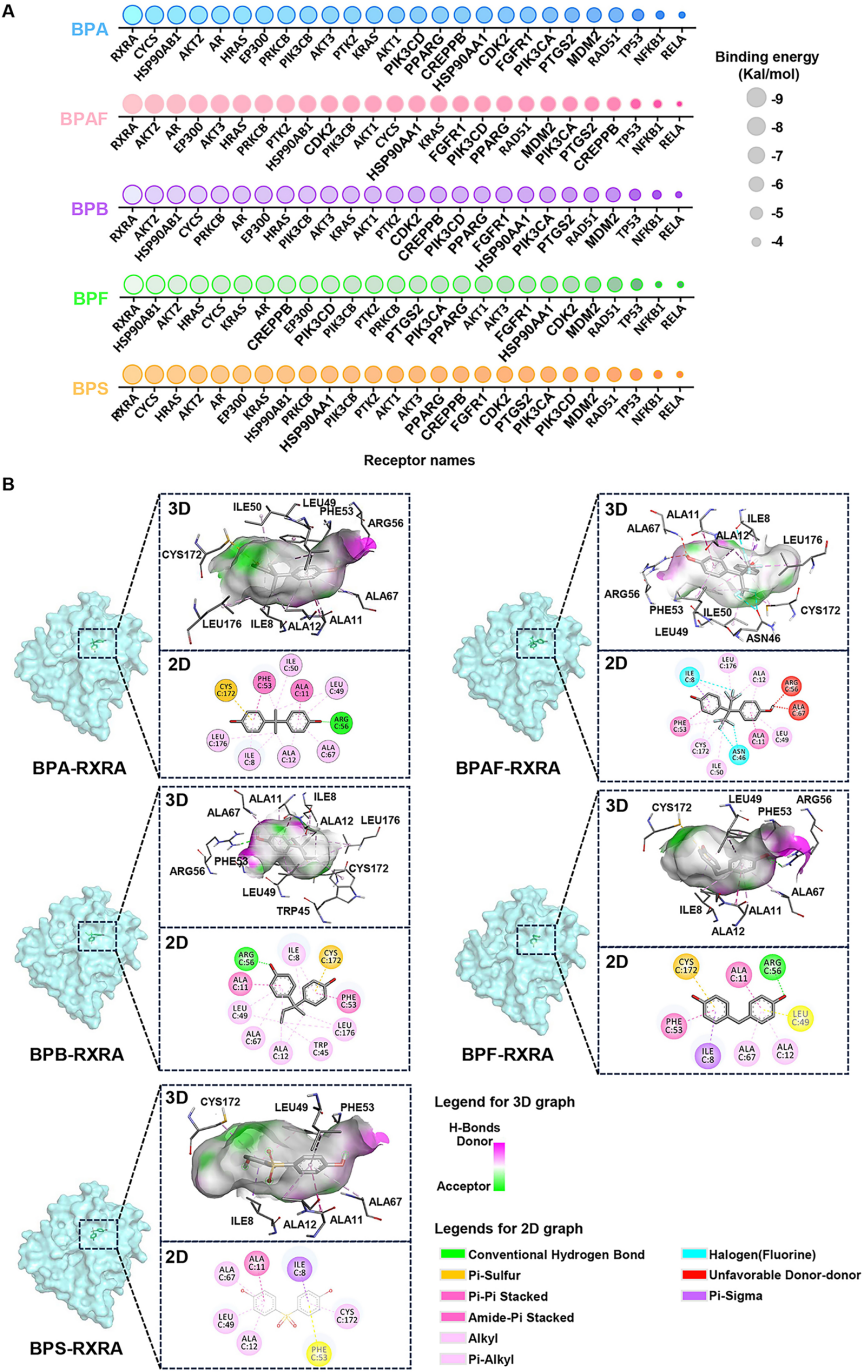
Molecular docking between BPs and core targets. **A** Bubble plot of binding energies between BPs and 26 core proteins. Circle size scales with binding energy magnitude. **B** Two-dimensional (2D) and three-dimensional (3D) schematic diagrams of molecular interactions for BPs and their core target RXRA

The hub target proteins were selected for molecular docking analysis with five BPA analogs to explore their potential binding modes. The multi-axis bubble heatmap indicated binding energies ranging from − 3.6 kcal/mol to -9.3 kcal/mol for the bisphenols (Fig. 4A). Notably, most hub target proteins exhibited binding energies below − 5 kcal/mol, signifying strong interactions with the bisphenols. All compounds showed the highest binding affinity with the RXRA (Retinoid X Receptor Alpha) protein, while the weakest interactions were observed with the RELA (Transcription factor p65) protein. The complex with the lowest binding energy formed between the bisphenols and RXRA was further analyzed to identify key binding interactions with pocket residues. As shown in Fig. 4B, all ligand-receptor complexes, except for BPAF, displayed stable hydrogen bond interactions, while BPAF exhibited stronger halogen interactions due to the trifluoromethyl groups. Additionally, residues Cys172, Phe53, Ile8, Ala67, Ala11, Arg56, Leu49, and Ala12 within the RXRA binding pocket were identified as critical for stabilizing the receptor-ligand complexes, suggesting that these bisphenol compounds share similar binding modes with endogenous target proteins.

### Molecular dynamics simulation analysis

**Fig. 5:**
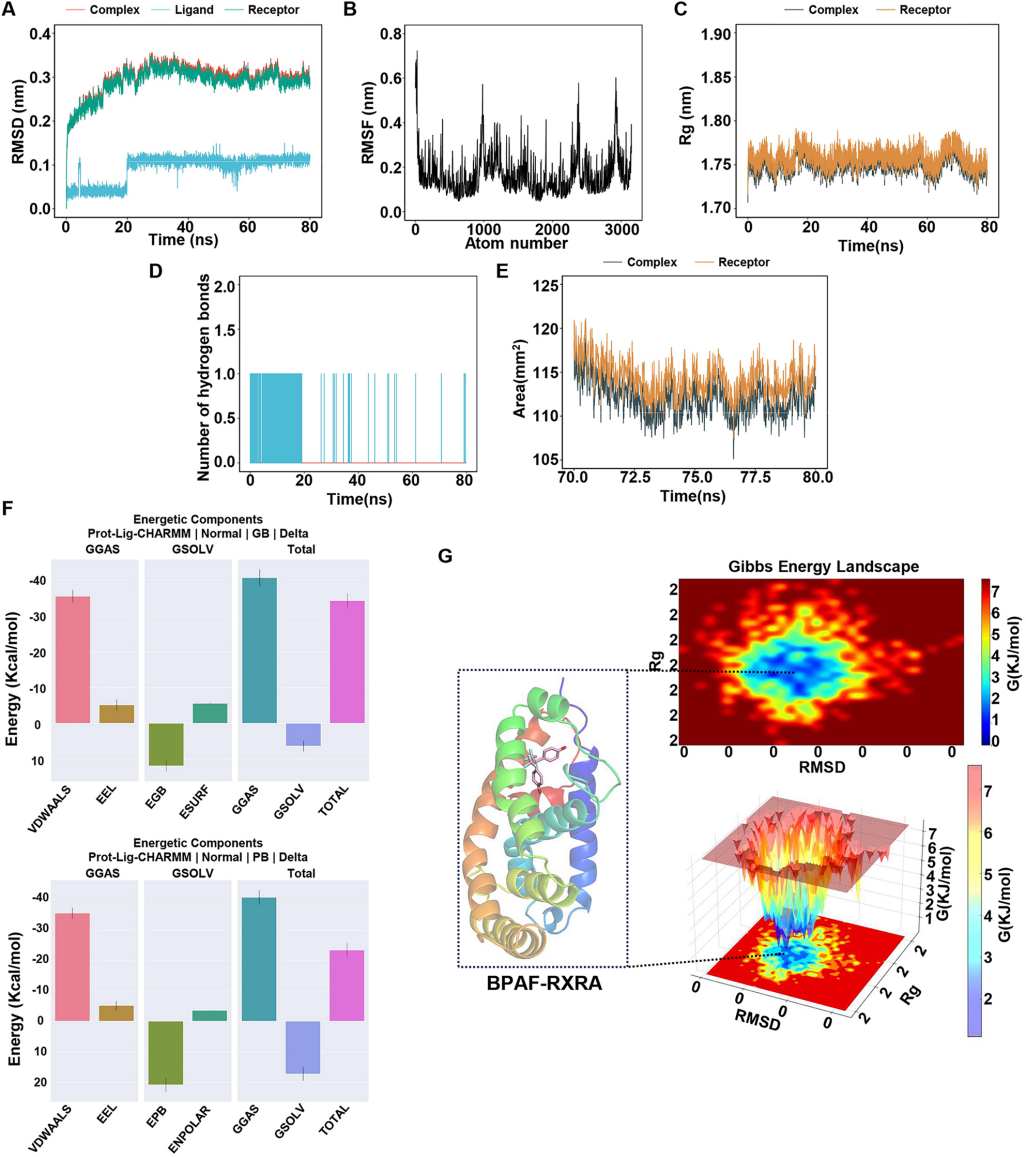
Molecular dynamics simulation trajectory analysis for the BPAF-RXRA complex. **A** RMSD plot. **B** RMSF plot. **C** Rg plot. **D** Number of hydrogen bonds plot. **E** SASA plot. **F** Decomposition of the total binding free energy into individual component contributions calculated by MM-GB/PBSA. **G** Two-dimensional (2D) and three-dimensional (3D) Gibbs free energy landscapes of the BPAF-RXRA complex, along with the dominant conformation diagram for the complex

To further assess the dynamic binding and stability, an 80 ns MD simulation was conducted on the BPAF-RXRA complex. Root Mean Square Deviation (RMSD) was used to evaluate the stability of the complex throughout the simulation. The BPAF-RXRA complex stabilized after 20 ns of fluctuations, with the RMSD values converging between 2.6 and 3.5 Å, indicating stable structural formation (Fig. 5A). Root Mean Square Fluctuation (RMSF) reflects the flexibility of amino acid residues during the simulation, and the results indicate that the RMSF values remained below 6 Å, except for the terminal residues, suggesting the receptor-ligand structure remained stable throughout the simulation (Fig. 5B). Radius of Gyration (Rg) characterizes the compactness and stability of the protein structure. As shown in Fig. 5C, the Rg value of RXRA significantly decreased upon binding with BPAF, indicating that ligand binding induced a more compact protein conformation. Hydrogen bonds are the primary non-covalent interactions responsible for stabilizing receptor-ligand complexes. Consistent with the molecular docking results, no stable hydrogen-bonding interactions were observed between RXRA and BPAF after post-equilibration (Fig. 5D). The SASA value, which quantifies protein surface exposure, reveals that RXRA underwent significant contraction upon binding with BPAF (Fig. 5E).

A stable 70–80 ns region of the MD trajectories was selected to calculate the binding free energy using the MM-PBSA/MM-GBSA approach, which reflects the influence of the solvent on the ligand-protein binding process. Figure 5F and Supplementary Table S2 present the calculated contributions of individual energy components and the mean binding free energies for the RXRA-BPAF complex system. The binding energy calculated using the MM-GBSA algorithm was − 34.32 kcal/mol, while the MM-PBSA algorithm yielded a value of -23.31 kcal/mol. These results indicate that BPAF exhibited favorable binding, as evidenced by the negative binding free energy values. The binding energy analysis showed favorable contributions from van der Waals (VDWAALS), electrostatic (EEL), surface area (ESURF), and nonpolar solvation (ENPOLAR), while polar solvation terms (EGB/EPB) contributed unfavorably.

In parallel, RMSD and Rg were utilized to characterize the Gibbs FEL (Free Energy Landscape), enabling visualization of binding free energy distributions and the identification of stable complex conformations from the MD simulations. Figure 5G displays the 2D and 3D free energy topography maps and the lowest-energy conformation derived from the 70–80 ns MD trajectory, with deep blue regions indicating the lowest energy states.

### Putative therapeutic interventions of TCM herbs and food targeting the bisphenol compounds exposure

**Fig. 6:**
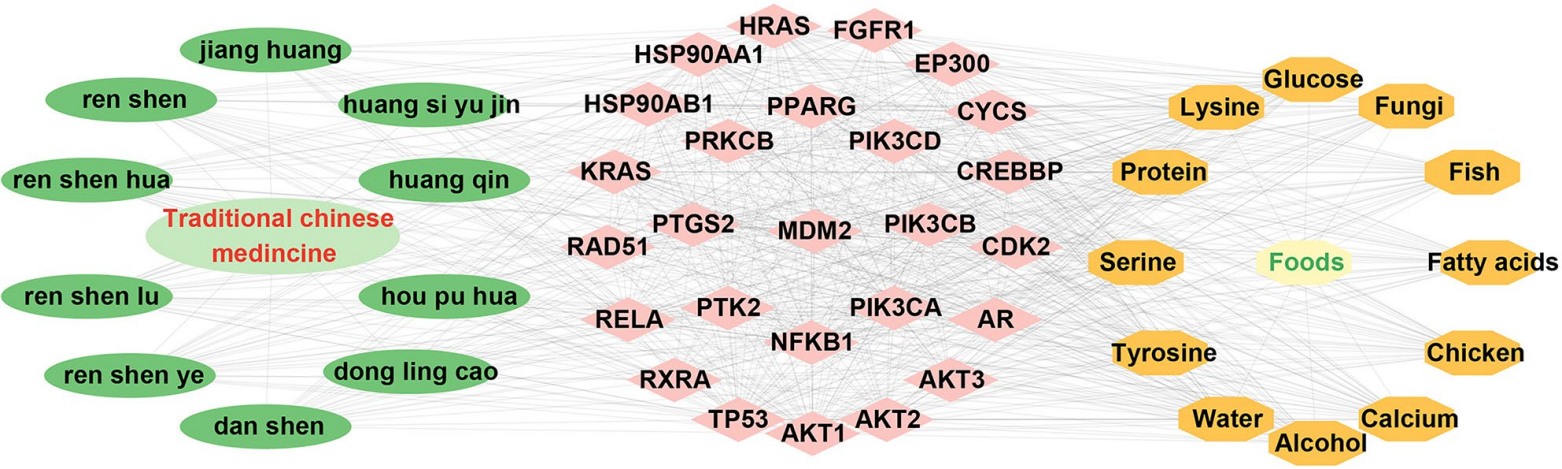
Network diagram of hub targets-TCM herbs-foods

Twenty-six hub targets were imported into the Coremine Medical database, and the top 10 ranked TCM herbs and foods were selected to construct a comprehensive interaction network among the hub targets, TCM herbs, and foods (Fig. 6). The network reveals key TCM herbs that can potentially mitigate cancer risk induced by bisphenols exposure, including *Ginseng (Ren shen)*,* Turmeric (Jiang huang)*,* Salvia miltiorrhiza (Dan shen)*,* Scutellaria baicalensis (Huang qin)*,* Magnolia officinalis (Hou pu)*,* Curcuma aromatica (Yu jin)*,* and Rabdosia rubescens (Dong lingcao)*. In TCM theory, these herbs primarily function to regulate Qi, clear heat, and resolve toxicity. Additionally, workers exposed to BPs should ensure adequate hydration, increase the intake of high-quality protein, calcium-rich foods, and incorporate fungi-rich foods.

### Analysis of the hub targets associated with bisphenol compound exposure in pan-cancer

**Fig. 7:**
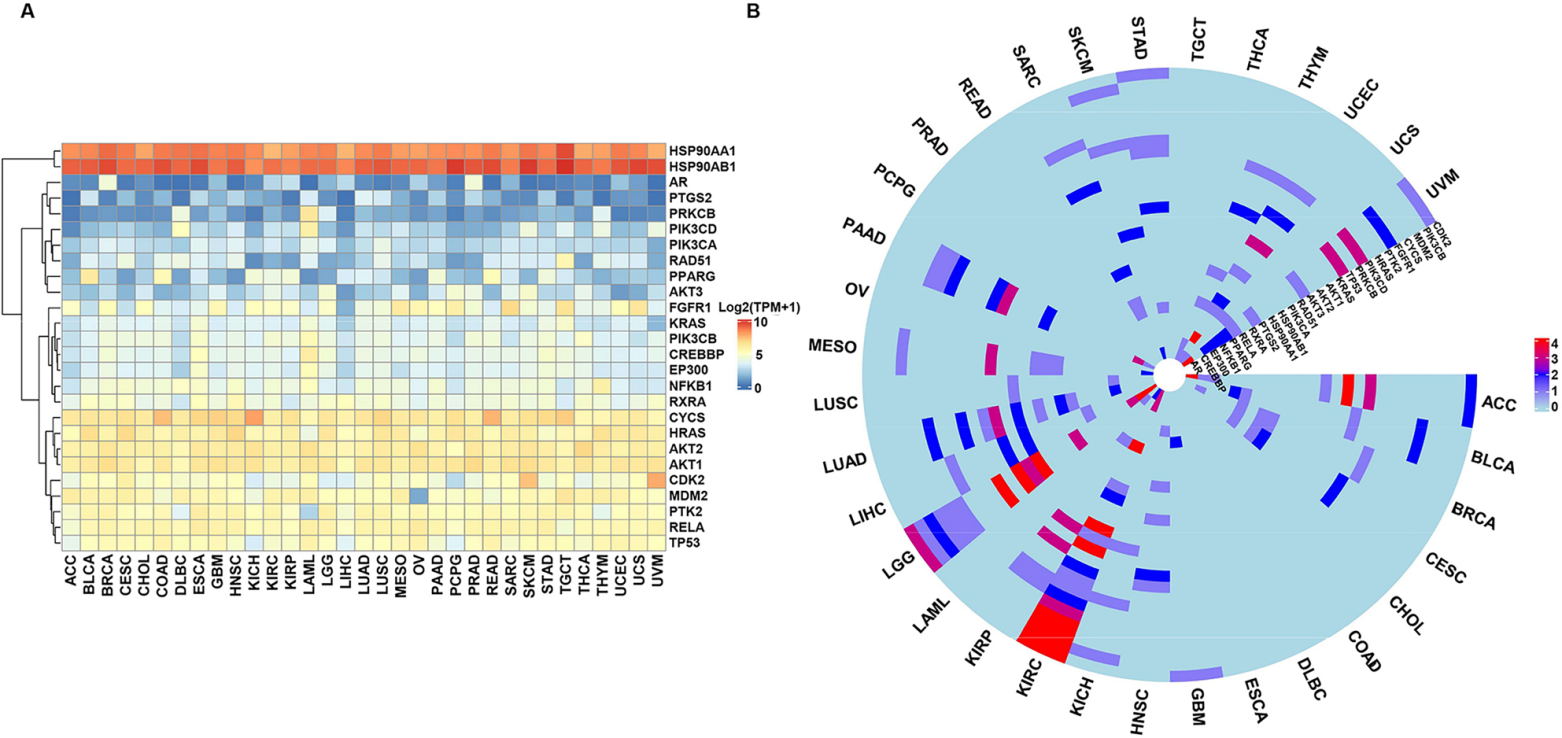
Pan-cancer analysis. **A** Heatmap showing the expression of core targets across pan-cancer types, with color intensity representing expression levels. The intensity scale ranges from blue (low) to red (high). **B** Circular heatmap of univariate Cox survival analysis for 26 bisphenol-induced tumorigenesis core targets across 33 cancers. Symbol encoding: “ns” for *P* > 0.05, “1” for *P* < 0.05, “2” for *P* < 0.01, “3” for *P* < 0.001, and “4” for *P* < 0.0001

To identify tumor types significantly associated with BPs exposure, the expression patterns and prognostic roles of the 26 hub targets were analyzed across 33 pan-cancer types. The heatmap results showed that HSP90AA1 (Heat Shock Protein 90 Alpha Family Class A Member 1) and HSP90AB1 (Heat Shock Protein 90 Alpha Family Class B Member 1) consistently exhibited high expression levels across all tumor types, while genes such as AR (Androgen Receptor), PTGS2 (Prostaglandin-Endoperoxide Synthase 2), PRKCB (Protein Kinase C Beta), PIK3CD (Phosphatidylinositol-4,5-Bisphosphate 3-Kinase Catalytic Subunit Delta), PIK3CA (Phosphatidylinositol-4,5-Bisphosphate 3-Kinase Catalytic Subunit Alpha), RAD51 (DNA repair protein RAD51 homolog 1), and PPARG (Peroxisome Proliferator-Activated Receptor Gamma) showed low expression in the majority of tumors (Fig. 7A). The circular heatmap demonstrated that the hub targets related to bisphenol exposure exhibited significant prognostic correlations in LGG and KIRC (Fig. 7B). Further studies focused on evaluating the diagnostic and prognostic value of these hub targets in these two cancer types to better understand the potential relationship between bisphenols exposure and tumor initiation, progression, and prognosis.

### Diagnostic model construction with six ensemble machine learning algorithms based on the hub targets associated with bisphenol compound exposure

**Fig. 8:**
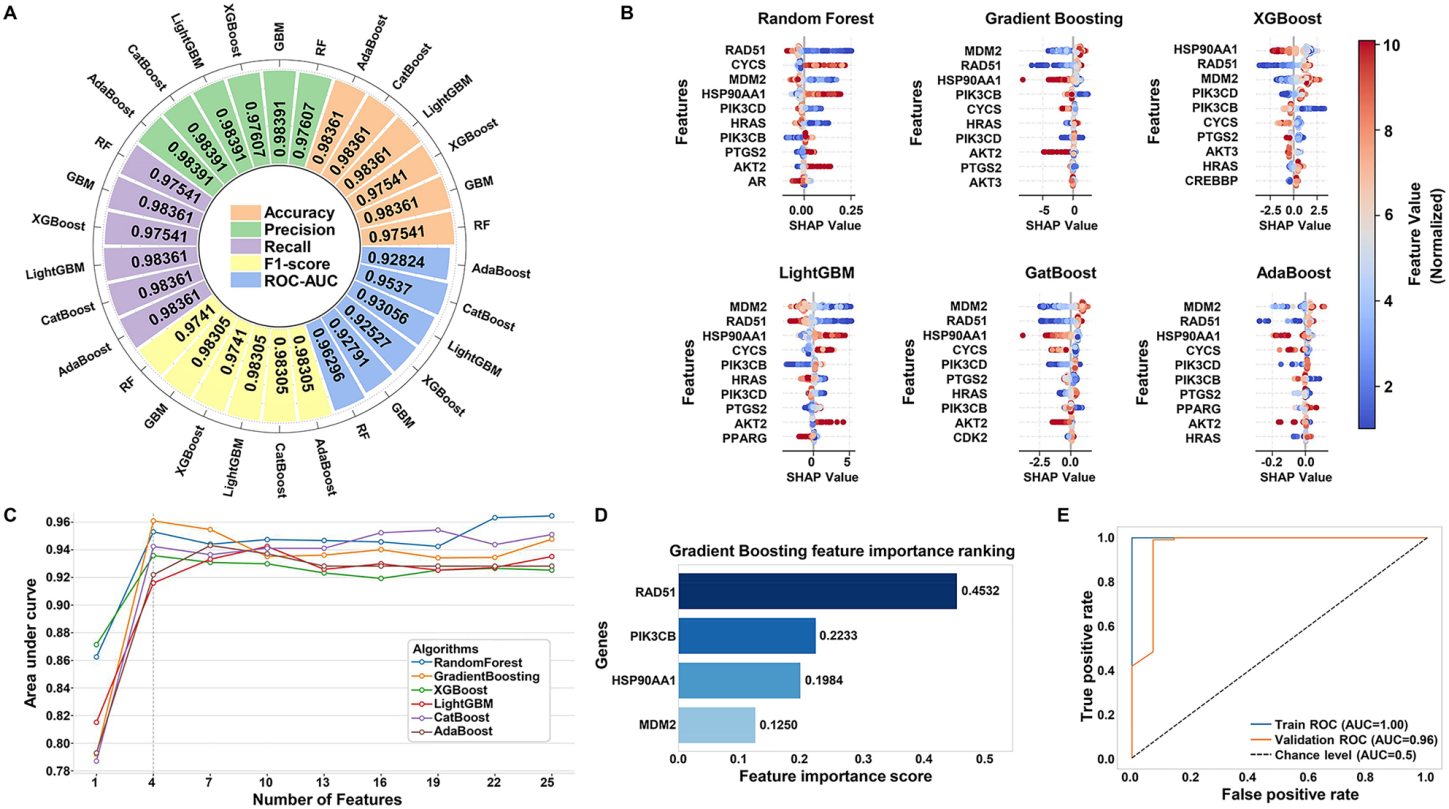
Construction of a diagnostic model based on core targets of BPs exposure-induced tumorigenesis in KIRC. **A** Performance evaluation of six ensemble machine learning models. ROC-AUC: Area under the curve of the receiver operating characteristic. **B** SHAP feature contribution ranking of core targets across six models. **C** Recursive feature selection based on AUC values. AUC: Area under the curve. **D** Bar plot of variable importance score based on the Gradient Boosting Machine. **E** Receiver Operating Characteristic (ROC) curves of the diagnostic model on training and validation datasets

Six ensemble machine learning models, including AdaBoost, GBM, LightGBM, RF, CatBoost, and XGBoost, were developed using 26 hub targets, and their performance was evaluated, as shown in Fig. 8A. All six models demonstrated exceptional performance, with accuracy, precision, recall, and the F1-scores exceeding 97%, and the ROC-AUC scores surpassing 0.9. These results indicate that the models possess strong predictive capabilities with reliable and robust classification outcomes. SHapley Additive exPlanations (SHAP) values were then calculated for the hub targets to quantify the contribution of each variable to the model predictions. Figure 8B presents the top 10 genes ranked by their contribution scores across the models, highlighting their critical roles in model interpretation. The results show minimal variation in the ranking of the top 10 genes across the six algorithms. Based on the SHAP-derived feature importance rankings and a recursive feature elimination approach, it was found that the GBM algorithm achieved the highest AUC value using just four features, indicating its superior predictive performance among the six models (Fig. 8C). The Gini impurity reduction-based feature importance ranking for GBM identified the top four discriminative features as RAD51, PIK3CB (Phosphatidylinositol 4,5-bisphosphate 3-kinase catalytic subunit beta isoform), HSP90AA1, and MDM2 (E3 ubiquitin-protein ligase Mdm2) (Fig. 8D). A refined GBM model trained on these genes achieved perfect classification in the training set (AUC = 1.0) and a 0.96 AUC on the test set, demonstrating excellent generalization and robust diagnostic performance (Fig. 8E).

### Development of prognostic risk models with the hub targets implicated in Bisphenol compounds exposure for KIRC

**Fig. 9:**
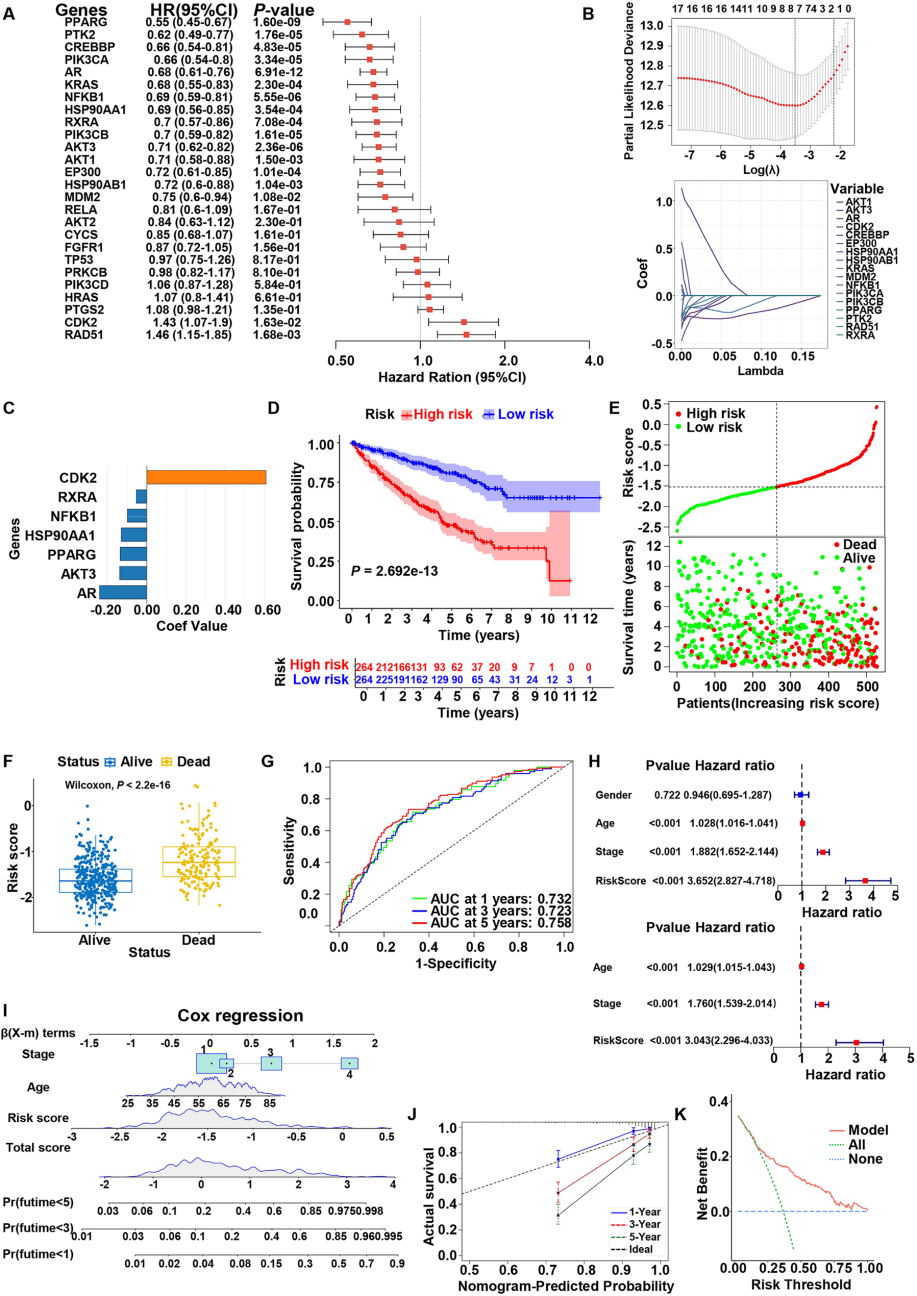
Construction of a prognostic risk model based on core targets of BPs exposure-induced tumorigenesis in KIRC. **A** Forest plot of univariate Cox regression analysis for the core targets. HR: hazard ratio, CI: confidence interval. **B** The least absolute shrinkage and selection operator (LASSO) Cox regression with core targets. **C** Coefficients of the 7 core targets. **D** Kaplan–Meier survival curves for overall survival in high- and low-risk groups. **E** The risk plot based on the prognostic risk model. The dashed line indicates the separation between low-risk and high-risk subgroups. **F** Box plot of survival status in high- and low-risk groups. **G** Time-dependent ROC curves based on the risk score. **H** Univariate and multivariate Cox regression of risk score and clinical features. **I** Nomogram model for predicting individual survival probabilities. **J** Calibration curve. **K** Decision curve analysis (DCA) plot

Univariate Cox regression analysis identified 17 genes among the 26 hub targets associated with BPs exposure that significantly correlated with overall survival (OS) in KIRC patients (*P* < 0.05) (Fig. 9A). To improve the model’s predictive accuracy, a LASSO-Cox proportional hazards regression model was constructed using these 17 genes to identify the optimal prognostic gene signature (Fig. 9B). Subsequently, 7 genes, shown in Fig. 9C, were selected to construct the KIRC prognostic model. The risk score (RS) for each patient was calculated as follows: RS = (−0.241 × AR) + (−0.098 × NFKB1) + (−0.135 × PPARG) + (−0.053 × RXRA) + (−0.131 × HSP90AA1) + (−0.138 × AKT3) + (0.608 × CDK2). The cohort was then divided into high-risk (*n* = 264) and low-risk (*n* = 264) subgroups using the median RS as the cutoff threshold. Kaplan–Meier survival analysis revealed that the low-risk group exhibited significantly better survival outcomes (*P* < 0.001) (Fig. 9D). The risk stratification plot demonstrated a higher mortality incidence in the high-risk cohort, with significantly elevated RSs in deceased patients compared to survivors (Fig. 9E and F). Time-dependent receiver operating characteristic (ROC) analysis demonstrated satisfactory discriminatory accuracy of the RS in predicting OS, with the AUC values of 0.732 at 1 year, 0.723 at 3 years, and 0.758 at 5 years, indicating strong sensitivity and specificity (Fig. 9G). Univariate and multivariate Cox proportional hazards regression analyses were performed to evaluate the prognostic impact of the RS alongside clinicopathological variables such as gender, age, and tumor stage. Univariate Cox regression analysis showed that the RS, age, and stage were significantly correlated with OS in KIRC patients. Multivariate Cox regression analysis further confirmed that RS, age, and tumor stage were independent prognostic factors for KIRC patients (Fig. 9H). Based on these independent prognostic factors, a nomogram was developed to predict 1-, 3-, and 5-year individual mortality for KIRC patients (Fig. 9I). Calibration curves indicated that the nomogram-based prediction was highly consistent with actual survival outcomes (Fig. 9J). Decision curve analysis (DCA) demonstrated that this nomogram-based prognostic model provided more net benefits for predicting patient prognosis (Fig. 9K).

### Constructing a prognostic model for LGG based on the hub targets of BPs exposure

**Fig. 10:**
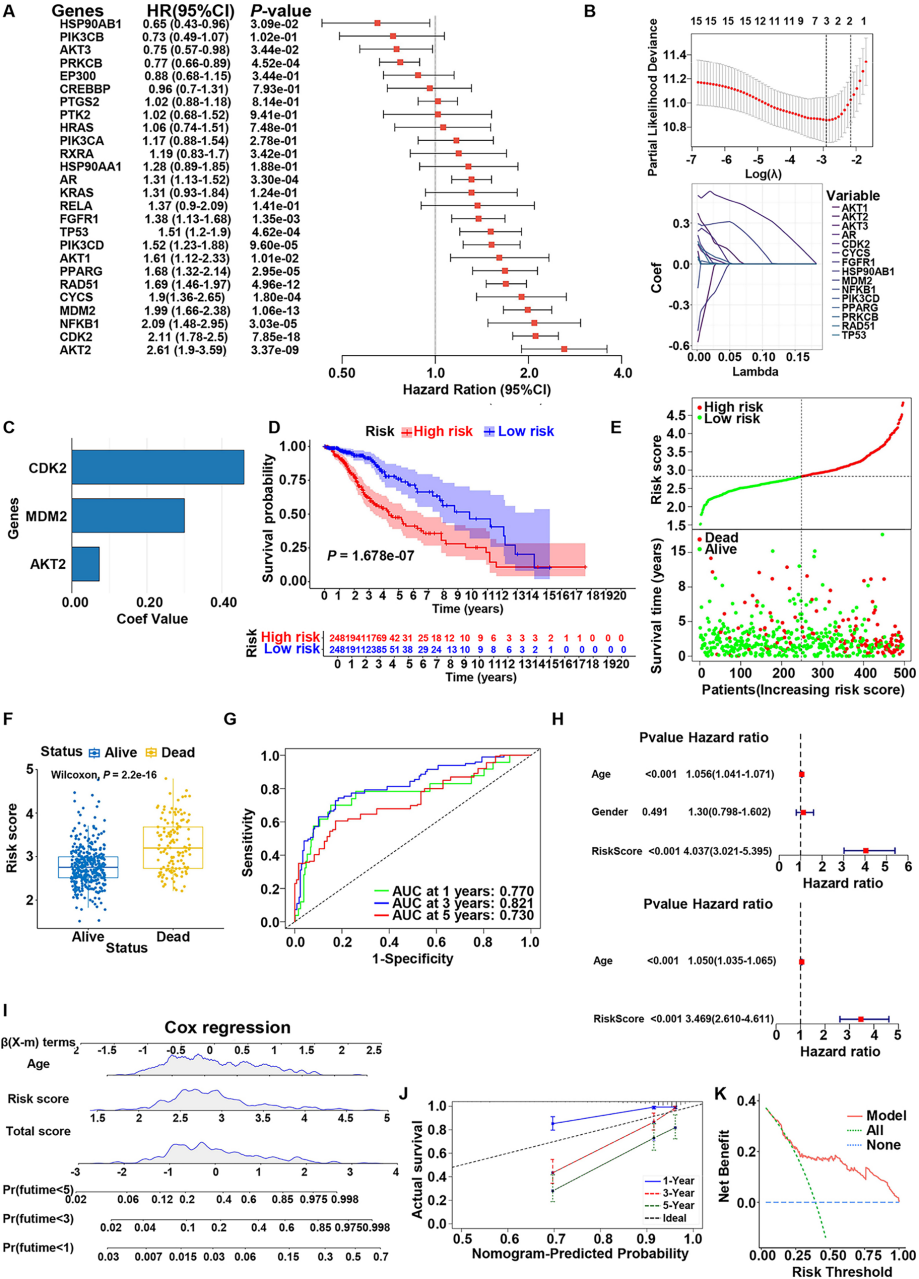
Construction of a prognostic risk model based on core targets of BPs exposure-induced tumorigenesis in LGG. **A** Forest plot of univariate Cox regression analysis for core targets. HR: hazard ratio, CI: confidence interval. **B** Least absolute shrinkage and selection operator (LASSO) Cox regression with core targets. **C** Coefficients of the 3 core targets. **D** Kaplan–Meier survival curves for high- and low-risk groups. **E** Risk plot based on the prognostic risk model. The dashed line separates low-risk and high-risk subgroups. **F** Box plot of survival status in high- and low-risk groups. **G** Time-dependent ROC curves based on the risk score. **H** Univariate and multivariate Cox regression of risk score and clinical features. **I** Nomogram model for predicting individual survival probabilities. **J** Calibration curve. **K** Decision curve analysis (DCA) plot

The prognostic significance of 26 hub targets associated with BPs exposure was also evaluated in LGG. Univariate Cox regression analysis revealed that the expression levels of 15 genes were significantly associated with OS in LGG patients (Fig. 10A). A Lasso-Cox regression model was constructed using these prognostically significant genes, with L1 regularization applied to mitigate overfitting and eliminate irrelevant genes (Fig. 10B). This approach resulted in a refined prognostic model incorporating three pivotal genes: CDK2 (Cyclin-dependent kinase 2), MDM2, and AKT2 (RAC-beta serine/threonine-protein kinase) (Fig. 10C). The RS for cancer patients were calculated by integrating the expression levels of these genes with their respective regression coefficients, using the formula: RS = (0.073 × AKT2) + (0.300 × MDM2) + (0.459 × CDK2). Based on the median risk score derived from the prognostic model, LGG patients were stratified into high-risk (*n* = 248) and low-risk (*n* = 248) groups. Kaplan–Meier survival analysis demonstrated that the low-risk group exhibited significantly better prognosis compared to the high-risk group (*P* < 0.001) (Fig. 10D). The high-risk group showed a markedly higher mortality rate, with deceased patients having significantly higher risk scores compared to survivors (Fig. 10E and F). Receiver Operating Characteristic curve analysis revealed that the risk score had promising potential in predicting 1-, 3-, and 5-year survival outcomes, with the time-dependent ROC analysis yielding AUC values of 0.770, 0.821, and 0.730, respectively (Fig. 10G). Univariate Cox regression analysis indicated that the risk score and age were significantly correlated with OS in LGG patients. Multivariate Cox analysis confirmed that RS and age were independent prognostic factors (Fig. 10H). To enhance clinical applicability, a nomogram model was developed incorporating RS and age (Fig. 10I). Calibration curves and decision curves demonstrated that the nomogram model exhibited strong predictive accuracy and significant clinical utility (Fig. 10J and K).

### Investigation of core modeling genes at the single-cell and protein levels

**Fig. 11:**
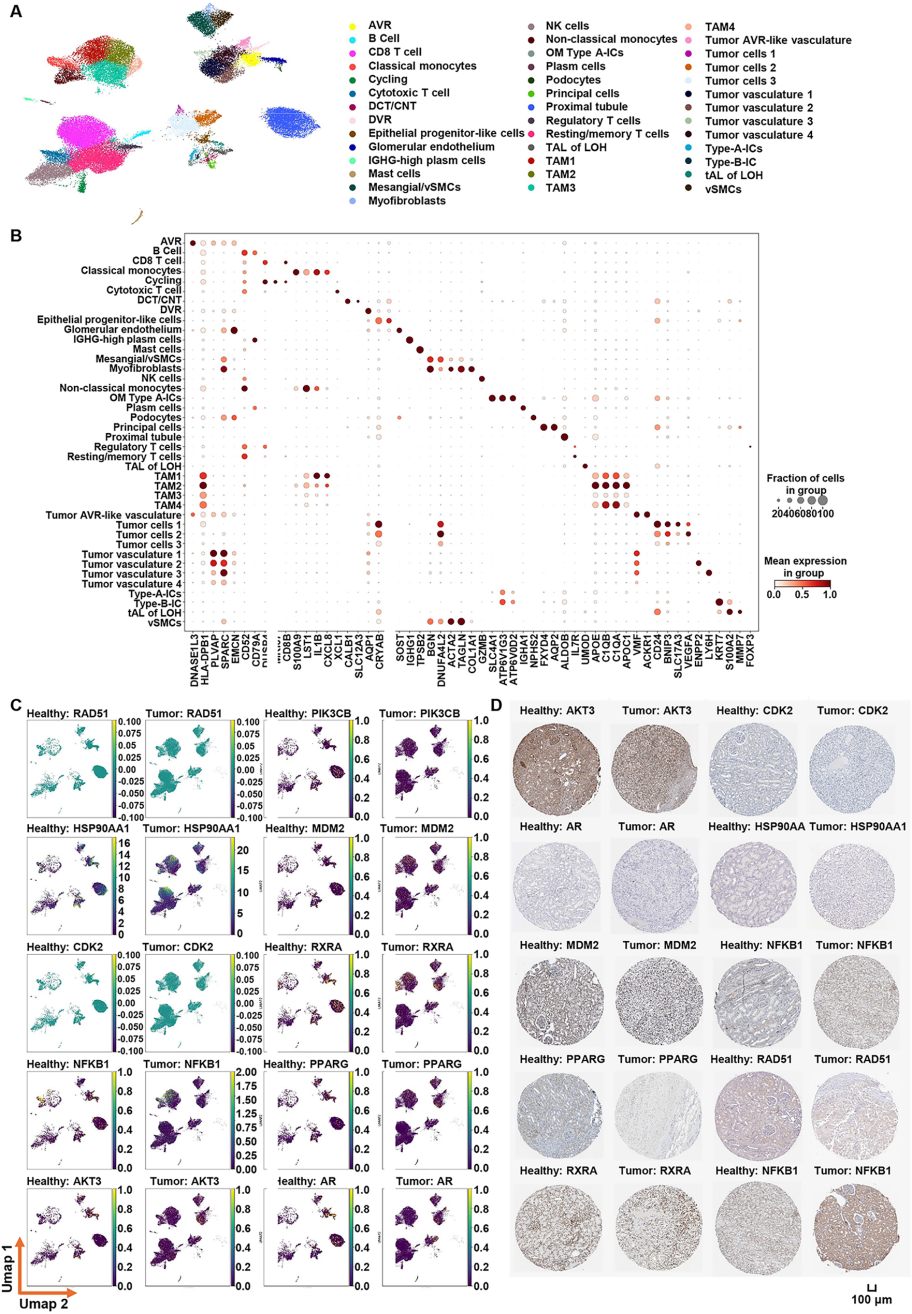
Single-cell sequencing analysis for KIRC. **A** Uniform Manifold Approximation and Projection (UMAP) visualization delineates distinct cell populations. **B** Bubble heatmap visualizes the distribution of canonical cell markers across diverse cellular subsets, where dot size corresponds to the proportion of expressing cells and color intensity reflects their average expression levels. **C** Uniform Manifold Approximation and Projection (UMAP) visualization depicting the expression landscape of core genes. **D** Immunohistochemistry staining (IHC) of core gene protein expression in normal and tumor tissues

To elucidate the potential biological roles of the core genes, their expression patterns across distinct cellular subpopulations were examined using scRNA-seq. After quality control, batch correction, and graph-based clustering, 40 distinct subpopulations were identified based on the expression patterns of canonical marker genes in KIRC (Fig. 11A and B). Feature map analysis revealed that the core genes modeled in KIRC—RAD51, PIK3CB, HSP90AA1, MDM2, CDK2, RXRA, NFKB1, PPARG, AKT3 (RAC-gamma serine/threonine-protein kinase), and AR—are predominantly expressed in tumor cell clusters and distinct immune cell subsets, including macrophages, CD8+ cytotoxic T cells, regulatory T cells (Tregs), NK cells, resting/memory T cells, plasma cells, myofibroblasts, and tumor-associated vascular endothelial cells (Fig. 11C). These findings suggest that the core genes identified in KIRC play pivotal roles in modulating the tumor immune microenvironment and promoting tumor angiogenesis, thereby contributing to KIRC initiation and progression. Immunohistochemistry staining (IHC) results from the Human Protein Atlas (HPA) database demonstrated that RAD51 and AR were significantly overexpressed in KIRC tumor tissues compared to healthy kidney tissues, while AKT3 showed reduced expression in tumors (Fig. 11D).

**Fig. 12:**
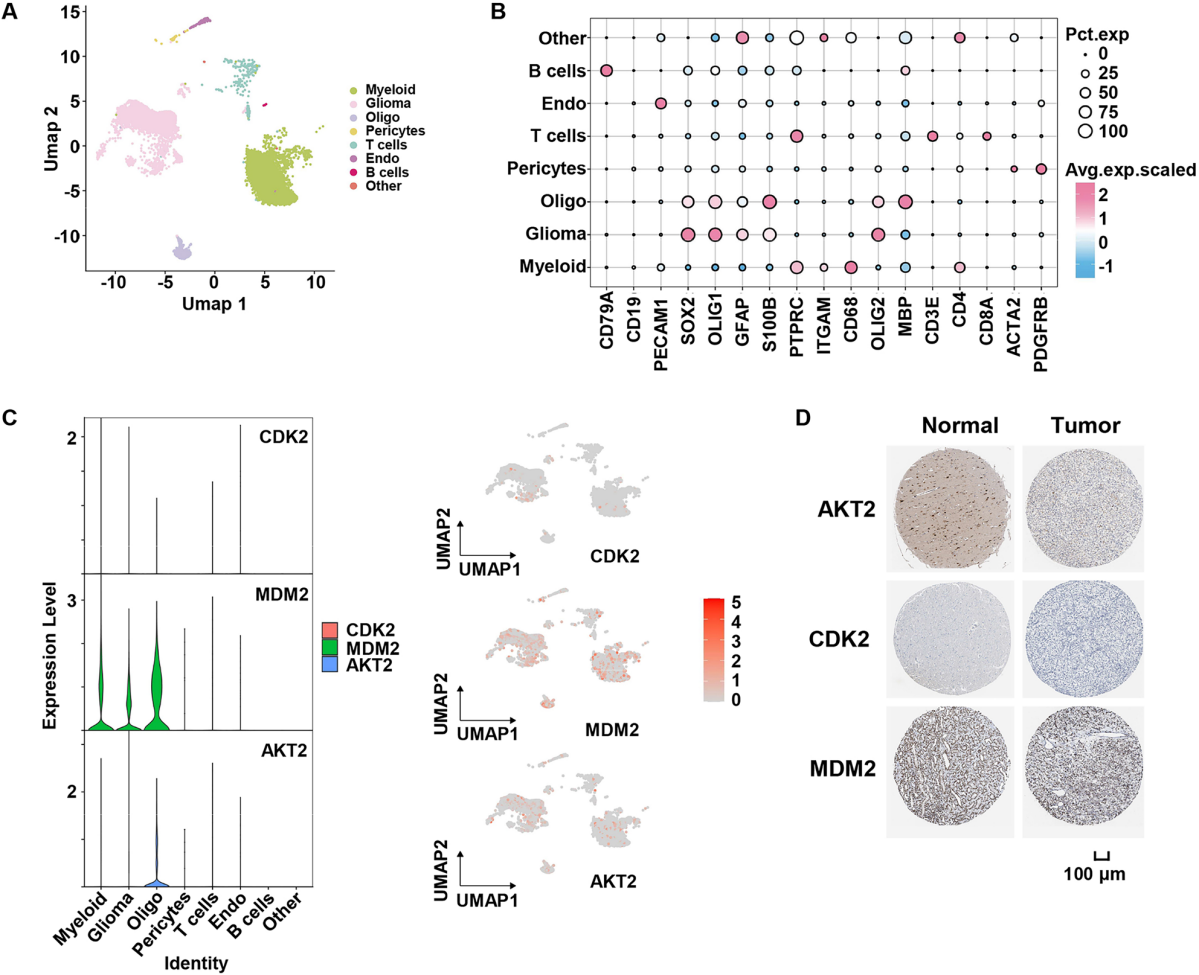
Single-cell sequencing analysis for LGG. **A** Uniform Manifold Approximation and Projection (UMAP) visualization delineates distinct cell populations. **B** Bubble heatmap visualizes the distribution of canonical cell markers across diverse cellular subsets, where dot size corresponds to the proportion of expressing cells and color intensity reflects their average expression levels. **C** Uniform Manifold Approximation and Projection (UMAP) visualization and violin plot visualization depicting the expression landscape of prognostic risk model-associated genes. **D** Immunohistochemical (IHC) staining of core gene protein expression in normal and tumor tissues

Similarly, unsupervised clustering using the Louvain community detection algorithm and marker-based annotation was performed on scRNA-seq data from LGG, resulting in the partitioning of the data into 8 distinct cell subpopulations (Fig. 12A and B). Feature map and violin plot analyses showed that the core genes CDK2, MDM2, and AKT2 were predominantly expressed in three cell subpopulations: myeloid cells, glioma cells, and oligodendrocytes (Fig. 12C). Additionally, IHC staining results demonstrated that AKT2 exhibited lower expression in LGG tumors compared to normal brain tissues (Fig. 12D).

## Discussion

Cancer is the second leading cause of death globally, and environmental exposures, such as EDCs, are known to influence cancer risk [22–24]. Bisphenols, among the most common EDCs, are increasingly detected in environmental waters, drinking water, and biological organisms at rising concentrations, thereby elevating human exposure risks [25]. The present study utilized a network toxicology approach to investigate the carcinogenic risks associated with exposure to five BPs and their underlying mechanisms. Initially, 1,904 potential targets of BPs were identified through retrieval from nine online databases. By intersecting these targets with tumor-related molecules, 1,112 BPs targets with potential carcinogenic risks were obtained. Functional and pathway enrichment analyses indicated that these genes are primarily involved in cellular responses to xenobiotic stimuli, chemical carcinogenesis, cell proliferation, and other functions. Specifically, the KEGG enrichment analysis highlighted strong associations between these targets and 14 types of cancer, including gastric, breast, and prostate cancers. Subsequently, a PPI network was constructed using the STRING database. Through calculation of network topological parameters, 26 core targets shared across the five BPs were identified. Molecular docking results revealed that, with the exception of TP53 (Cellular tumor antigen p53), NFKB1 (Nuclear factor NF-kappa-B p105 subunit), and RELA, the binding energies between BPs and the other core targets were all below −5 kcal/mol, indicating strong binding affinity between BPs and most core targets. This suggests that BPs may interfere with target functions through stable binding, thus promoting carcinogenic processes. Notably, RXRA exhibited the lowest binding energy with the five BPs. Retinoid X receptor alpha (RXRA), a member of the non-steroidal nuclear receptor superfamily, exerts pro-oncogenic functions in various tumor cells through its N-terminally truncated isoform (tRXRα). The primary mechanisms include activation of PI3K/AKT and NF-κB signaling pathways [26, 27]. Analysis of the Coremine Medical database revealed that traditional Chinese medicines such as *Ginseng (Ren shen)*, *Turmeric (Jiang huang)*, *Salvia miltiorrhiza (Dan shen)*, *Scutellaria baicalensis (Huang qin)*, *Magnolia officinalis (Hou pu)*, *Curcuma aromatica (Yu jin)*, and *Rabdosia rubescens (Dong lingcao)* may help mitigate the carcinogenic risks associated with BPs exposure. In summary, BPs exposure appears to activate multiple signaling pathways, including those involved in cell proliferation, chemical carcinogenesis, and oxidative stress, thereby increasing the risk of cancer development.

To identify tumor types most associated with BPs exposure, survival analysis was conducted on 26 core targets across 33 tumor types from the TCGA database, using median expression levels as the threshold. The results indicated that LGG and KIRC exhibited the strongest associations with BPs exposure. Six ensemble machine learning algorithms, combined with SHAP value analysis, were then employed to identify four hub genes (RAD51, PIK3CB, HSP90AA1, and MDM2) in KIRC. These genes were used to construct a diagnostic model, which demonstrated excellent predictive performance, with the AUC values of 1.0 in the training set and 0.96 in the validation set. To further explore the prognostic value of these core targets, a risk scoring model was developed using seven key genes (AR, NFKB1, PPARG, RXRA, HSP90AA1, AKT3, and CDK2), based on feature selection via LASSO-Cox proportional hazards regression. The results showed that high-risk patients had poorer OS outcomes. Additionally, the risk score served as an independent prognostic factor for KIRC, and a nomogram based on it effectively predicted patient survival probabilities. Since the LGG datasets exclusively consist of tumor samples, a prognostic model incorporating CDK2, MDM2, and AKT2 was constructed using the same methodology applied to KIRC. Kaplan–Meier survival analysis revealed significantly better prognosis in the low-risk group compared to the high-risk group.

Subsequent scRNA-seq analysis of KIRC revealed that the core genes used in model construction were predominantly expressed in immune-related cells, including macrophages, CD8+ cytotoxic T cells, Tregs, NK cells, resting/memory T cells, plasma cells, and myofibroblasts. This observation suggests that BPs exposure may increase tumorigenesis risk by disrupting the immune microenvironment. Notably, IHC analysis revealed significantly elevated protein levels of RAD51 and AR in tumor tissues compared to normal kidney tissues. DNA repair protein RAD51 homolog 1 (RAD51) plays a pivotal role in DNA repair via homologous recombination. In KIRC, RAD51 expression, both at the protein and mRNA levels, serves as a prognostic biomarker for survival, with higher expression correlating with significantly poorer prognosis outcomes [28]. Elevated AR expression at both the transcriptomic and proteomic levels consistently correlated with improved OS in KIRC patients. Additionally, AR modulates the circHIAT1/miR-195-5p/29a-3p/29c-3p/CDC42 signaling pathway, promoting KIRC cell migration and invasion [29, 30]. In contrast, in LGG, the prognostic model genes were primarily expressed in myeloid, glioma, and oligodendrocyte cell subpopulations. These findings suggest that BPs exposure may contribute to tumorigenesis by disrupting the intrinsic properties of glial cells. Concurrently, significantly elevated protein levels of AKT2 were observed in glioma tissues compared to normal tissues. RAC-beta serine/threonine-protein kinase (AKT2), an isoform of AKT, plays a critical role in the tumorigenesis of various human cancers. Elevated AKT2 expression is positively correlated with malignant progression in gliomas, and silencing AKT2 expression suppresses glioma cell migration and invasion by reducing MMP2 (72 kDa type IV collagenase) and MMP9 (Matrix metalloproteinase-9) levels [31, 32]. These results demonstrate that BPs exposure increases carcinogenic risk by modulating the immune microenvironment and altering key protein expression levels involved in cancer progression.

However, several limitations are present in this study. First, while the current evidence linking BPs exposure to increased cancer risk is largely based on data mining, machine learning, and bioinformatics, direct experimental validation to confirm causality is lacking. Second, the findings may be influenced by algorithmic biases, data quality, and source variability. Third, the core targets mediating BPs-induced carcinogenesis have been validated computationally for binding stability solely through molecular docking and dynamics simulations. Further experimental confirmation using advanced biophysical techniques—including Thermal Proteome Profiling (TPP), Surface Plasmon Resonance (SPR), Cellular Thermal Shift Assay (CETSA), and Atomic Force Microscopy (AFM)—is necessary to validate molecular interactions and clarify the mechanistic details of BPs-induced carcinogenesis. Finally, future research will aim to combine experimental validation with prospective epidemiological studies to provide direct evidence of BPs’ contribution to cancer risk.

## Conclusion

This study employed a comprehensive pan-cancer analysis, integrating network toxicology, bioinformatics, machine learning, molecular docking, and molecular dynamics (MD) simulations, to elucidate the core targets and underlying mechanisms of the putative carcinogenic risks associated with BPs. The findings demonstrate that BPs increase cancer susceptibility by modulating multiple signaling pathways involved in cellular responses to xenobiotic stimuli, chemical carcinogenesis, and the regulation of cell proliferation. Notably, pan-cancer analysis revealed significant associations between BPs exposure and the pathogenesis of KIRC and LGG. To mitigate health risks associated with BPs exposure, it is strongly recommended to prioritize the use of glass or stainless-steel products over BPs-containing plastic utensils and water bottles. Additionally, traditional Chinese medicine and dietary interventions are proposed as potential strategies for mitigating these risks. The research provides valuable insights into BPs-related carcinogenesis and future targeted therapeutic approaches, offering an innovative framework for studying health risks arising from environmental pollutant exposure.

## Supplementary Information

Below is the link to the electronic supplementary material.

Supplementary Material 1

## Data Availability

All data supporting the findings of this study are available within the paper and its Supplementary Information.

## Abbreviations

AdaBoost: Adaptive Boosting
ADMET: Absorption, distribution, metabolism, excretion, and toxicity
AKT2: RAC-beta serine/threonine-protein kinase
AKT3: RAC-gamma serine/threonine-protein kinase
AR: Androgen Receptor
AUC: Area under the curve
BP: Biological processe
BPA: Bisphenol A
BPAF: Bisphenol AF
BPB: Bisphenol B
BPF: Bisphenol F
BPs: Bisphenols
BPS: Bisphenol S
CatBoost: Categorical Boosting
CDH1: Cadherin-1
CDK2: Cyclin-dependent kinase 2
CI: Confidence interval
CYCS: Cytochrome c
DCA: Decision curve analysis
EDCs: Endocrine-disrupting chemicals
EEL: Electrostatic
EGB/EPB: Polar solvation terms
ENPOLAR: Nonpolar solvation
ESURF: Surface area
FEL: Free Energy Landscape
GBM: Gradient Boosting Machine
GEO: Gene Expression Omnibus
GO: Gene Ontology
HCC: Hepatocellular carcinoma
HPLC-MS/MS: High-performance liquid chromatography coupled with mass spectrometry
HR: Hazard ratio
HSP90AA1: Heat Shock Protein 90 Alpha Family Class A Member 1
HSP90AB1: Heat Shock Protein 90 Alpha Family Class B Member 1
IGC50: 50% growth inhibition concentration in Tetrahymena pyriformis
IHC: Immunohistochemistry
KEGG: Kyoto Encyclopedia of Genes and Genomes
KIRC: Kidney renal clear cell carcinoma
KM: Kaplan–Meier
Lasso-Cox: Least Absolute Shrinkage and Selection Operator Cox proportional hazards model
LC50DM: Median lethal concentration in Daphnia magna
LC50FM: Median lethal concentration in Pimephales promelas
LGG: Low-grade glioma
LightGBM: Light Gradient Boosting Machine
Log P: Octanol-water partition coefficient
MD: Molecular dynamics
MDM2: E3 ubiquitin-protein ligase Mdm2
MF: Molecular function
MM/GBSA: Molecular Mechanics/Generalized Born Surface Area
MM/PBSA: Molecular Mechanics/Poisson-Boltzmann Surface Area
MMP2: 72 kDa type IV collagenase
MMP9: Matrix metalloproteinase-9
Mt.: Molecular weight
NFKB1: Nuclear factor NF-kappa-B p105 subunit
NHANES: National Health and Nutrition Examination Survey
NPT: Constant pressure and temperature
NVT: Constant temperature and volume
OS: Overall survival
PC: Polycarbonate
PIK3CA: Phosphatidylinositol-4,5-Bisphosphate 3-Kinase Catalytic Subunit Alpha
PIK3CB: Phosphatidylinositol 4,5-bisphosphate 3-kinase catalytic subunit beta isoform
PIK3CD: Phosphatidylinositol-4,5-bisphosphate 3-kinase catalytic subunit delta
PPARG: Peroxisome Proliferator-Activated Receptor Gamma
PPI: Protein-protein interaction
PPO: Polyphenylene oxide
PRKCB: Protein Kinase C Beta
PSU: Polysulfone
PTGS2: Prostaglandin-Endoperoxide Synthase 2
RAD51: DNA repair protein RAD51 homolog 1
RELA: Transcription factor p65
RF: Random Forest
Rg: Radius of Gyration
RMSD: Root Mean Square Deviation
RMSF: Root Mean Square Fluctuation
ROC: Receiver operating characteristic curve
RS: Risk score
RXRA: Retinoid X Receptor Alpha
SASA: Solvent Accessible Surface Area
scRNA-seq: Single-cell RNA sequencing
SDF: Structure Data File
SFN: 14-3-3 protein sigma
SHAP: SHapley Additive exPlanations
TCGA: The Cancer Genome Atlas
TCM: Traditional Chinese Medicine
TNFRSF10C: Tumor necrosis factor receptor superfamily member 10C
TP53: Cellular tumor antigen p53
UMAP: Uniform Manifold Approximation and Projection
VDWAALS: Van der Waals
XGBoost: Extreme Gradient Boosting
