# Supplementary material for: Unraveling the potential carcinogenic risk of bisphenols: a comprehensive network analysis and computational toxicology insights
Source: Hum Genomics. 2026 Mar 14;20:75. doi: 10.1186/s40246-026-00948-5 (PMC13101310; doi:10.1186/s40246-026-00948-5)
Supplement: Supplementary file 1 — Supplementary Material 1 [file 40246_2026_948_MOESM1_ESM.docx]

**Supplementary tables**

**Table S1.** Basic information on five types of Bisphenol Compounds.

| Name | Abbreviation | SMILES | Molecular  Formula | Molecular  Weight | CAS |
| --- | --- | --- | --- | --- | --- |
| Bisphenol A | BPA | CC(C)(C1=CC=C(C=C1)O)C2=CC=C(C=C2)O | C_15_H_16_O_2_ | 228.29 | 80-05-7 |
| Bisphenol B | BPB | CCC(C)(C1=CC=C(C=C1)O)C2=CC=C(C=C2)O | C_16_H_18_O_2_ | 242.31 | 77-40-7 |
| Bisphenol F | BPF | C1=CC(=CC=C1CC2=CC=C(C=C2)O)O | C_13_H_12_O_2_ | 200.23 | 620-92-8 |
| Bisphenol S | BPS | C1=CC(=CC=C1O)S(=O)(=O)C2=CC=C(C=C2)O | C_12_H_10_O_4_S | 250.27 | 80-09-1 |
| Bisphenol AF | BPAF | C1=CC(=CC=C1C(C2=CC=C(C=C2)O)(C(F)(F)F)C(F)(F)F)O | C_15_H_10_F_6_O_2_ | 336.23 | 1478-61-1 |

**Table S2.** Binding free energy and energy decomposition terms calculated using the MMGBSA and MMPBSA methods.

| **Calculation method** | **VDWAALS** | **EEL** | **EGB** | **ESURF** | **GGAS** | **GSOLV** | **TOTAL** |
| --- | --- | --- | --- | --- | --- | --- | --- |
| MMGBSA | -35.51±1.04 | -5.17±0.52 | 11.9±0.02 | -5.55±0.03 | -40.67±1.36 | 6.35±0.04 | -34.32±1.36 |
|  | **VDWAALS** | **EEL** | **EPB** | **ENPOLAR** | **GGAS** | **GSOLV** | **TOTAL** |
| MMPBSA | -35.51±1.04 | -5.17±0.52 | 20.92±0.07 | -3.56±0.02 | -40.67±1.36 | 17.36±0.07 | -23.31±1.36 |
